# Trans-orbital sonography of the optic nerve in multiple sclerosis

**DOI:** 10.1371/journal.pone.0336564

**Published:** 2025-11-17

**Authors:** Seyedehnarges Tabatabaee, Negin Eissazade, Zahra Mirzaasgari, Seyed Mosa Tabatabaee, Leila Raeesmohammadi, Mohammad Reza Motamed

**Affiliations:** 1 Department of Neurology, School of Medicine, Iran University of Medical Sciences, Tehran, Iran; 2 Cellular and Molecular Research Center, Iran University of Medical Sciences, Tehran, Iran; 3 Brain and Cognition Clinic, Institute for Cognitive Sciences Studies, Tehran, Iran; 4 Faculty Member of Cognitive Sciences, Department of Psychology and Educational Sciences, Semnan University, Semnan, Iran; Creighton University, UNITED STATES OF AMERICA

## Abstract

**Background:**

Trans-orbital sonography (TOS) has emerged as a non-invasive tool for detecting optic nerve damage by measuring optic nerve diameter (OND) and optic nerve sheath diameter (ONSD).

**Objective:**

This study aimed to assess OND, ONSD, and their ratio in patients with MS and healthy controls, and to explore their potential associations with clinical and paraclinical parameters.

**Methods:**

In this comparative cross-sectional study (January 2022-September 2022), we enrolled adult (≥ 18 years) patients with MS, diagnosed based on the 2017 revised McDonald criteria, who were on stable disease-modifying drugs, and healthy volunteers without neurological or ophthalmic conditions. Sonographic assessments of OND and ONSD were performed using an M-Turbo ultrasound machine with an 8-MHz linear probe.

**Results:**

A total of 56 patients with MS and 60 healthy controls were included. OND, ONSD, and OND/ONSD were significantly smaller in patients with MS, even after adjusting for age (p < 0.001). The reductions in OND and ONSD were more pronounced in individuals with a history of optic neuritis, while the OND/ONSD ratio remained unaffected by op. No significant associations were observed between OND or ONSD values and age, sex, MS type, EDSS score, disease duration, DMD, or cervical or thoracic cord lesions. However, infratentorial lesions were associated with smaller right ONSD (p = 0.017).

**Conclusions:**

Reduced OND and ONSD in patients with MS, especially in those with prior optic neuritis, suggest that sonographic evaluation may reflect subclinical optic nerve atrophy. These findings support the utility of OND and ONSD as potential structural markers in MS, though further longitudinal and multimodal studies are needed to confirm their diagnostic and prognostic value.

## Introduction

Optic neuritis (ON) affects roughly half of patients with multiple sclerosis (MS) over the course of disease [1] and typically presents with subacute, unilateral vision loss, driven by inflammation-induced retinal nerve fiber layer (RNFL) thinning and axonal loss [2–4]. However, RNFL thinning can also occur in the absence of clinically apparent ON, plausibly due to damage within the optic radiations (geniculocalcarine tract) or inflammation involving other vulnerable segments of the afferent visual pathway, such as the myelinated optic nerves and chiasma [2–5].

Previous studies have reported significantly reduced optic nerve diameter (OND) and optic nerve sheath diameter (ONSD) in MS [6–8]. Although advanced modalities, including optical coherence tomography (OCT), visual evoked potentials (VEP), and magnetic resonance imaging (MRI), are widely used to detect visual dysfunction and degeneration of the retina and optic nerve, there is growing interest in bedside, non-invasive, and cost-effective alternatives [5,9,10]. Trans‑orbital sonography (TOS) has emerged as a potential surrogate marker of neurodegeneration, as it allows for direct measurement of OND and ONSD, typically at 3 [7,9,11–15] and 5 mm [7,13–15] posterior to the globe [16], and has shown promise for detecting both acute ON and chronic atrophy. Nevertheless, conclusive evidence regarding its validity and reliability remains limited.

Given this background, we measured OND and ONSD in patients with MS, with and without prior ON, and in healthy controls, to assess the diagnostic potential of TOS while disentangling ON-related injury from ON-independent neurodegeneration.

## Materials and methods

### Study design and participants

This comparative cross-sectional study was conducted in Firoozgar Hospital, affiliated with Iran University of Medical Sciences, between January 15, 2022 and September 15, 2022.

The study protocol was approved by the Ethics Committee of Iran University of Medical Sciences (IR.IUMS.FMD.REC.1400.561). Written informed consent was obtained from all participants, and all procedures adhered to relevant ethical guidelines. Data were de-identified (pseudonymized) before analysis to protect confidentiality, and only the research team had access to the dataset.

Convenience sampling was used to enroll eligible adults (≥ 18 years), with a confirmed diagnosis of MS according to the 2017 revised McDonald criteria [17], who were receiving disease-modifying drugs (DMDs) [18], along with healthy volunteers serving as controls. Exclusion criteria were relapse or acute neurological events (e.g., ON within the previous three months) (to avoid acute inflammatory swelling of the optic nerve and sheath [8,9,12,13]); any change in DMD regimen during the preceding year; major systemic disorders (e.g., diabetes mellitus, hypertension) or other conditions known to affect the optic nerve; neurological diseases other than MS; significant history of head trauma or ocular injury; ophthalmic conditions such as glaucoma or a history of ocular surgery; and pregnancy at the time of assessment.

Controls were medical students and staff, with no history of neurological, psychiatric, ophtalmic, or systemic disorder, that could affect the optic nerve or visual pathway.

### Data collection and tools

Each participant underwent comprehensive laboratory testing to exclude systemic disorders, along with thorough general, ophthalmic, and neurological examinations. We collected demographics, disease duration, current DMDs, Kurtzke Expanded Disability Status Scale (EDSS) [19] scores, and MRI data.

A neuroradiologist reviewed the gadolinium-enhanced brain and cervical/thoracic spine MRIs (1.5-T; Siemens Healthineers, Erlangen, Germany) to rule out active inflammatory lesions; the number and location of lesions were then reported based on T2-weighted images [20]. Lesion counts were categorized into four groups: < 7, 7–15, 16–25 and > 25 [8,21].

A board-certified neurologist, with 10 years of clinical experience in neuroimaging and ocular sonography, blinded to baseline clinical data and group assignment, performed TOS on all participants using an M-Turbo ultrasound machine with an 8-MHz linear probe (FUJIFILM SonoSite., Bothell, WA, USA) to minimize inter-observer variability. Gain and depth settings were standardized across participants.

Participants were positioned supine with 20–30° head elevation. After gel application to the temporal side of the closed upper eyelid, the anterior optic nerve was imaged transversely, visualizing the hypoechoic optic nerve 3 mm posterior to the globe. The ONSD appeared as thin bilateral hyperechoic lines surrounding the hypoechoic optic nerve [14,22]. OND and ONSD were each measured in triplicate, and averages were used for analysis.

### Sample size and statistical analysis

Assuming a two-sided α = 0.05, 80% power, a 10–15% attrition rate, the required sample size was 16 participants per group [8]. Patients with MS were categorized into two subgroups: those with a history of optic neuritis (MS-ON) and those without a history optic neuritis (MS-NON).

The Kolmogorov-Smirnov test was used to assess normality. Categorical variables are presented as frequency and percentage, and continuous variables as mean ± standard deviation. Categorical variables were compared using the Chi-square test. Correlations between continuous variables were assessed using Pearson’s correlation coefficient for normally distributed data and Spearman’s rank correlation coefficient otherwise. For group comparisons of continuous outcomes, we used the independent-samples t-test or one-way analysis of variance (one-way ANOVA) for normally distributed variables, and the Mann-Whitney U test and Kruskal-Wallis test for non-normally distributed variables. We conducted a one-way multivariate analysis of covariance (MANCOVA) to compare OND, ONSD, and the OND/ONSD ratio between MS and control groups, adjusting for age as a covariate; model assumptions were examined and met.

All analyses were performed via IBM SPSS Statistics, version 27, with statistical significance set at *p*-value < 0.05.

## Results and discussion

A total of 56 patients with MS (32 females (57.1%); mean age: 40.32 ± 9.12 years) (Table 1) and 60 healthy controls (35 females (58.3%); mean age: 31.68 ± 8.47 years) were included. Sex distribution did not differ between groups (p = 0.90), but patients with MS were significantly older than controls (p < 0.001).

**Table 1:** Comparison of the baseline characteristics of the participants.

	MS-ON (n = 24)	MS-NON (n = 32)	*p*-value	Total (n = 56)
**Sex (female)**	13 (54.2%)	19 (59.4%)	0.3	32 (57.10%)
**Age (years)**	38.83 ± 9.4	41.44 ± 8.9	0.34	40.32 ± 9.12
**MS type**				
SPMS	15 (62.5%)	23 (71.9%)	0.24	38 (67.9%)
RRMS	9 (37.5%)	7 (21.9%)		16 (28.6%)
PPMS	–	2 (6.3%)		2 (3.6%)
**EDSS score**				
< 2	11 (45.8%)	9 (28.1%)	0.17	20 (35.7%)
≥ 2	13 (54.2%)	23 (71.9%)		36 (64.3%)
**Disease duration (years)**				
< 1	–	1 (3.1%)	0.38	1 (1.8%)
1-5	2 (8.3%)	7 (21.9%)		9 (16.1%)
6-10	9 (37.5%)	13 (40.6%)		22 (39.3%)
11-15	8 (33.3%)	5 (15.6%)		13 (23.2%)
> 15	5 (20.8%)	6 (18.8%)		11 (19.6%)
**Family history of MS**	7 (29.2%)	12 (37.5%)	0.51	19 (33.9%)
**Disease-modifying drugs**				
Rituximab	15 (62.5%)	28 (87.5%)	0.09	43 (76.8%)
Fingolimod	4 (16.7%)	1 (3.1%)		5 (8.9%)
Interferon beta-1b	2 (8.3%)	3 (9.4%)		5 (8.9%)
Glatiramer acetate	2 (8.3%)	–		2 (3.6%)
Natalizumab	1 (4.2%)	–		1 (1.8%)
**Number of lesions**				
< 7	–	–	0.34	–
7-15	5 (20.8%)	12 (37.5%)		17 (30.4%)
16-25	12 (50.0%)	12 (37.5%)		24 (42.9%)
> 25	7 (29.2%)	8 (25.0%)		15 (26.8%)
**Lesion distribution**				
Cervical cord	24 (100%)	30 (93.8%)	0.21	54 (96.4%)
Thoracic cord	2 (8.3%)	6 (18.8%)	0.27	8 (14.3%)
Infratentorial lesions	12 (50.0%)	20 (62.5%)	0.35	32 (57.1%)

EDSS: Expanded Disability Status Scale; PPMS: Primary Progressive Multiple Sclerosis; RRMS: Relapsing-Remitting Multiple Sclerosis; SPMS: Secondary Progressive Multiple Sclerosis.

*Categorical variables were compared using the Chi-square test, and means were compared using the Mann-Whitney U test.

Across the MS cohort, OND, ONSD, and the OND/ONSD ratio were not significantly associated with sex, age, MS type, EDSS score, disease duration, family history of MS, DMDs, number of lesions, or cervical/thoracic lesions. Infratentorial lesions were associated with smaller right ONSD (p = 0.017). A history of right-sided ON was associated with smaller right OND and ONSD (both p < 0.001), and a history of left-sided ON was associated with smaller left OND (p < 0.001) and ONSD (p = 0.002) (Table 2).

**Table 2:** Relationships between OND, ONSD, and OND/ONSD ratio, and demographic, clinical and paraclinical characteristics of patients with MS (p-value).

	Rt OND	Lt OND	Rt ONSD	Lt ONSD	Rt OND/ONSD	Lt OND/ONSD
**Demographics**						
**Sex**	0.44 ^t^	0.13	0.57 ^t^	0.40	0.67 ^t^	0.33 ^t^
**Age**	0.19	0.78	0.89	0.97	0.09	0.87
**Disease characteristics**						
**MS type**	0.21 ^a^	0.37	0.38 ^a^	0.41	0.61 ^a^	0.93 ^a^
**EDSS score**	0.09 ^t^	0.87	0.15	0.82	0.27 ^t^	0.79
**Disease duration**	0.89 ^a^	0.57	0.91	0.25	0.75 ^a^	0.35
**Family history of MS**	0.45 ^t^	0.71 ^t^	0.59 ^t^	0.91	0.69 ^t^	0.79 ^t^
**DMDs**	0.59 ^a^	0.53	0.59	0.25	0.33 ^a^	0.34 ^a^
**Number of lesions**	0.58 ^a^	0.13 ^a^	0.98	0.65	0.32 ^a^	0.13
**Lesion distribution**						
**Cervical cord**	0.98	0.55	0.69	0.81	0.77 ^t^	0.41
**Thoracic cord**	0.72	0.55	0.13	0.31	0.31 ^t^	0.09
**Infratentorial**	0.24	0.91	**0.017**	0.37	0.76 ^t^	0.34
**History of ON**						
**Right eye**	**< 0.001** ^t^	0.47	**< 0.001**	0.14	0.13 ^t^	0.29 ^t^
**Left eye**	0.49 ^t^	**< 0.001**	0.24 ^t^	**0.002**	0.89	0.23

DMDs: Disease-Modifying Drugs; EDSS: Expanded Disability Status Scale; MS: Multiple Sclerosis; OND: Optic Nerve Diameter; ONSD: Optic Nerve Sheath Diameter; ON: Optic Neuritis.

OND, ONSD, and the OND/ONSD ratio on both sides were significantly smaller in patients with MS, compared to healthy controls, even after adjusting for age (p < 0.001). The reductions in OND and ONSD were particularly pronounced in those with prior ON; however, OND/ONSD ratio did not significantly differ between patients with MS with and without prior ON (Table 3).

**Table 3:** Comparison of OND, ONSD, and OND/ONSD ratio between patients with MS with and without a history of ON, and healthy controls.

	MS-ON (n = 24)	MS-NON (n = 32)	*p*-value	Total (n = 56)	Control (n = 60)	*p*-value
**OND (mm)**						
Right	2.46 ± 0.37	2.84 ± 0.29	**< 0.001**	2.68 ± 0.37	3.95 ± 0.56	**< 0.001** ^**t**^
Left	2.59 ± 0.28	2.83 ± 0.25	**0.001** ^**t**^	2.73 ± 0.29	3.94 ± 0.50	**< 0.001** ^**t**^
**ONSD (mm)**						
Right	4.78 ± 0.49	5.23 ± 0.45	**< 0.001**	5.04 ± 0.51	5.39 ± 0.55	**0.001**
Left	4.82 ± 0.47	5.24 ± 0.49	**0.006**	5.06 ± 0.52	5.41 ± 0.51	**< 0.001**
**OND/ONSD**						
Right	0.52 ± 0.06	0.54 ± 0.06	0.09 ^t^	0.53 ± 0.06	0.73 ± 0.07	**< 0.001** ^**t**^
Left	0.54 ± 0.05	0.54 ± 0.07	0.51	0.54 ± 0.06	0.73 ± 0.07	**< 0.001**

OND: Optic Nerve Diameter; ONSD: Optic Nerve Sheath Diameter.

Our findings indicate that relapse-free patients with MS exhibit significantly reduced OND and ONSD compared with healthy controls. Several studies likewise report significantly smaller OND [6,8,9,15,23], ONSD [7,8,13–15,23], and OND/ONSD ratio [8] in MS. One study also found a smaller ONSD (5 mm posterior to the globe) in SPMS compared with those with RRMS [7], suggesting that optic nerve atrophy may progress with disease severity. In contrast, two studies observed no significant differences [12,24]. Furthermore, in a study including patients with MS and neuromyelitis optica spectrum disorder (NMOSD)—AQP4-positive/negative and MOG-positive)—OND and ONSD did not significantly differ between the groups [25].

Consistent with previous reports [11,13,23,25], we found that a history of ON is associated with smaller OND and ONSD, a pattern compatible with subclinical inflammation and/or diffuse axonal degeneration leading to structural optic nerve injury in MS. However, multiple investigations did not observe a consistent association between ON history and reductions in OND or ONSD [6,8,9,12,24]. Timing likely contributes to these discrepancies: in patients with MS with first-episode retrobulbar neuritis (n = 20), the OND of the affected eye was 0.6 mm greater than that of the unaffected eye [21], suggesting acute swelling may mask the chronic atrophy seen later.

We found that infratentorial lesions were associated with a smaller right ONSD, whereas lesions in the cervical and thoracic spinal cord showed no such relationship. This localization-specific pattern may reflect broader neuroanatomical connectivity: lesions near the visual pathways could influence CSF dynamics [26], thereby altering the thickness of the optic nerve subarachnoid space [27]. This interpretation is consistent with prior evidence linking ONSD to intracranial CSF pressure [27,28]. Moreover, infratentorial involvement has been associated with greater disability [29] and autonomic dysregulation [30–32], which can affect venous outflow and CSF flow, potentially contributing to ONSD alterations. Finally, subtle anatomical asymmetries in the optic canal or nerve [33,34] may accentuate side-specific effects in regions with altered CSF compliance.

In our cohort, TOS-derived measurements did not vary by sex, age, MS type, EDSS score, disease duration, family history of MS, or use of DMDs. By contrast, several studies have linked smaller OND [6,8,12] and ONSD [6,8,13,14] to higher EDSS scores, reflecting greater disability. ONSD has also been reported to correlate with disease duration [13], MRI-detected lesions [13,21], P100 amplitude [13], and P100 latency [8,13]; similarly, OND has been associated with disease duration [6], normalized total brain volume, gray matter volume, white matter volume, and ventricular CSF volume [9]. These discrepancies likely reflect heterogeneity in sample sizes and participant characteristics, disease duration, sonographic protocols, diagnostic thresholds, and imaging timing.

Despite promising findings, several limitations warrant caution. Convenience sampling in a tertiary-referral setting likely contributed to an atypical female-to-male ratio, MS type mix, and a high proportion on rituximab (> 75%); while reflective of local practice, the small single-center sample, and variability in DMD regimens constrain generalizability. Patients were significantly older than healthy controls; although age was adjusted for, residual confounding may have persisted. Controls were drawn from medical students and staff, which may not represent the general population. Normative OND and ONSD values were unavailable, limiting comparisons to established standards. The cross-sectional design prevents causal inference or assessment of temporal change. TOS captures structural changes; integration with functional measures (e.g., VEPs) would strengthen structure-function correlations. All ultrasounds were performed by a single trained neurologist, which reduces inter-observer variability yet underscores operator dependence and potential reproducibility challenges. Nearly all patients had cervical cord lesions, suggesting possible selection bias toward more severe cases, and exclusion of comorbidities further narrows applicability. Future longitudinal, multicenter studies with larger, more diverse cohorts and multimodal assessments are needed to validate and extend these results.

## Conclusions

TOS detected optic nerve alterations in MS, particularly among individuals with prior ON. Although these findings support TOS as a potentially informative adjunct for assessing disease burden, the cross-sectional design and modest sample size limit causal inference and immediate clinical translation. Larger prospective studies are needed to validate these observations and to define the role of TOS as a complementary tool in MS assessments.

## Supporting information

S1 Data This file includes the raw data used to generate the study’s results. (XLSX)
